# Volumetric Reductions of Subcortical Structures and Their Localizations in Alcohol-Dependent Patients

**DOI:** 10.3389/fneur.2019.00247

**Published:** 2019-03-19

**Authors:** Jae-Hyuk Shim, Yong-Tae Kim, Siekyeong Kim, Hyeon-Man Baek

**Affiliations:** ^1^Department of Health Sciences and Technology, GAIHST, Gachon University, Incheon, South Korea; ^2^Department of Psychiatry, College of Medicine, Chungbuk National University, Cheongju, South Korea

**Keywords:** MRI, subcortical, alcohol dependence, 3T, FSL, vertex analysis, FIRST

## Abstract

Changes in brain morphometry have been extensively reported in various studies examining the effects of chronic alcohol use in alcohol-dependent patients. Such studies were able to confirm the association between chronic alcohol use and volumetric reductions in subcortical structures using FSL (FMRIB software library). However, each study that utilized FSL had different sets of subcortical structures that showed significant volumetric reduction. First, we aimed to investigate the reproducibility of using FSL to assess volumetric differences of subcortical structures between alcohol-dependent patients and control subjects. Second, we aimed to use Vertex analysis, a less utilized program, to visually inspect 3D meshes of subcortical structures and observe significant shape abnormalities that occurred in each subcortical structure. Vertex analysis results from the hippocampus and thalamus were overlaid on top of their respective subregional atlases to further pinpoint the subregional locations where shape abnormalities occurred. We analyzed the volumes of 14 subcortical structures (bilateral thalamus, caudate, putamen, globus pallidus, hippocampus, amygdala, nucleus accumbens) in 21 alcohol-dependent subjects and 21 healthy controls using images acquired with 3T MRI. The images were run through various programs found in FSL, such as SIENAX, FIRST, and Vertex analysis. We found that in alcohol-dependent patients, the bilateral thalamus (left: *p* < 0.01, right: *p* = 0.01), bilateral putamen (left: *p* = 0.02, right: *p* < 0.01), right globus pallidus (*p* < 0.01), bilateral hippocampus (left: *p* = 0.05, right: *p* = 0.03) and bilateral nucleus accumbens (left: *p* = 0.05, right: *p* = 0.03) were significantly reduced compared to the corresponding subcortical structures of healthy controls. With vertex analysis, we observed surface reductions of the following hippocampal subfields: Presubiculum, hippocampal tail, hippocampal molecular layer, hippocampal fissure, fimbria, and CA3. We reproduced the assessment made in previous studies that reductions in subcortical volume were negatively associated with alcohol dependence by using the FMRIB Software Library. In addition, we identified the subfields of the thalamus and hippocampus that showed volumetric reduction.

## Introduction

While alcohol abuse is a prominent health concern worldwide, it is particularly a more severe problem in South Korea, where social pressures and situations often force people to drink, especially in corporate settings. Due to such pressures, statistics suggest that 55.3% of Korean males over the age of 19 consume dangerous amount of alcohol at least once per month (described by a bottle of soju or 5 cans of beer per sitting) and 21.2% Korean males over the age of 19 consume dangerous amounts of alcohol regularly (1, 2). As such, South Korean males are very likely to develop dependence on alcohol due to constant exposure to alcohol, and withdrawal symptoms make it very difficult to stop its misuse. In order to better understand how withdrawal symptoms arise from persistent alcohol misuse, it is important to examine how alcohol abuse affects brain structures in alcohol-dependent patients.

Patients with alcohol dependency are known to show symptoms such as craving, shaking, sweating, hallucinations, depression, insomnia, and anxiety. Various studies were able to correlate such symptoms to issues or activations within subcortical brain structures. Cravings of alcohol and similar drugs were linked to the thalamus (3), amygdala (4, 5) and the nucleus accumbens (6), while anxiety and depression were linked to the hippocampus (7) and amygdala (4). Insomnia has been linked to atrophic changes in the hippocampus and putamen (8). While it is unclear if such symptoms are directly related to damage caused by alcohol abuse, there have been investigations on how each subcortical structure changes under consistent exposure to alcohol.

Reduction of brain structures in alcohol-dependent individuals has been a common theme in various studies that examine the effects of chronic alcohol use on the brain. Reductions of white and gray matter (9) as well as in subcortical regions such as the hippocampus, nucleus accumbens (10), putamen and the caudate (11) have been reported in patients with alcohol dependence. It has been accepted that factors such as oxidative stress, inflammatory cytokines, and thiamine deficiency are major contributors to reductions in brain structures due to chronic alcohol consumption. It has also been considered that certain portions of the brain experience different rates of degeneration in chronic exposure to alcohol depending on how each structure reacts to loss of nutrition and to inhibition of neuroregeneration (12).

However, each of the various studies observing the differences in the volumes of subcortical structures between alcohol-dependent patients and healthy controls presented different variations of subcortical structures that showed significant reductions. Due to these inconsistencies, we present our own data on which subcortical structures showed volumetric reductions when comparing subcortical structures of alcohol-dependent patients and healthy controls. Our data provide additional information on how alcohol dependence affects each subcortical structure and provide insight on how they can be related to symptoms displayed by alcohol-dependent patients.

To identify volumetric changes of subcortical structures between alcohol-dependent patients and healthy controls, we utilized the FMRIB Software Library (FSL), a collection of programs used to automatically segment subcortical structures from 3T MRI images (13). Previous studies have used FSL to compare subcortical volumes automatically and consistently to show that subcortical structures in alcohol-dependent patients are reduced compared to subcortical structures in healthy controls. We aimed to investigate the reproducibility of using FSL to find similar volumetric reductions in subcortical structures of alcohol-dependent patients (13, 14). Due to the small size of subcortical structures, it is vital that an automated program can consistently distinguish volumetric differences between patients and controls. In addition, we utilized FSL's vertex analysis, which uses a design matrix to test for significant group differences (*p* < 0.05) between subcortical volumes and displays its results on the surface of each compared subcortical structure. We utilized this feature to localize each volumetric reduction on to a specific location on the surface of each affected substructure to further pinpoint where each subcortical structure experiences deterioration due to alcohol abuse. The orange surface layers designating significant shape abnormalities generated from the vertex analysis of the hippocampus and thalamus were overlaid on top of their respective subregional atlases to identify the specific subregions that experienced deterioration.

## Materials and Methods

### Participants

A total of 42 right-handed male subjects were involved in this study. Twenty-one patients suffering from alcohol dependence were recruited from a mental hospital specializing in alcohol. Twenty-one age-matched (range 40–63 years, mean 50.4 years) healthy controls were recruited by advertisement. All subjects were interviewed by a psychiatrist using the Diagnostic Statistical Manual 4th Edition (DSM-IV), which determined that each recruited patient met the criteria for alcohol dependence (one of which includes the manifestation of withdrawal symptoms). Recruited healthy controls did not meet the criteria for alcohol dependence. All participants with a history of a mental disorder, neurologic disease, psychiatric disorders, and other substance use disorders (excluding nicotine and caffeine) were excluded from the study. The Korean version of the Alcohol Use Disorder Identification Test (AUDIT-K) (11) and the Short Michigan Alcoholism Screening Test (SMAST) (15) were applied to all subjects. AUDIT-K and SMAST are both brief questionnaires aimed at self-assessment of alcohol dependence, with higher scores indicating high risk of alcohol dependence. The study protocol was approved by the Bioethics Committee Board of Chungbuk National University.

### Image Acquisition

All structural images were collected using a 3T Philips Achieva Scanner (Philips Medical System, Best, Netherlands) at Ochang campus of Korean Basic Science Institute. All subjects were scanned with the same 32-channel head coil at the same pulse sequence. MRI T1 parameters were referenced from the Alzheimer's Disease Neuroimaging Initiative (ADNI) protocol (TR = 6.8 ms, TE = 3.1 ms, field of view = 256 mm, flip angle = 9°, voxel size = 1 × 1 × 1.2 mm, 170 slices without gaps).

### Image Processing

**Figure 1:**
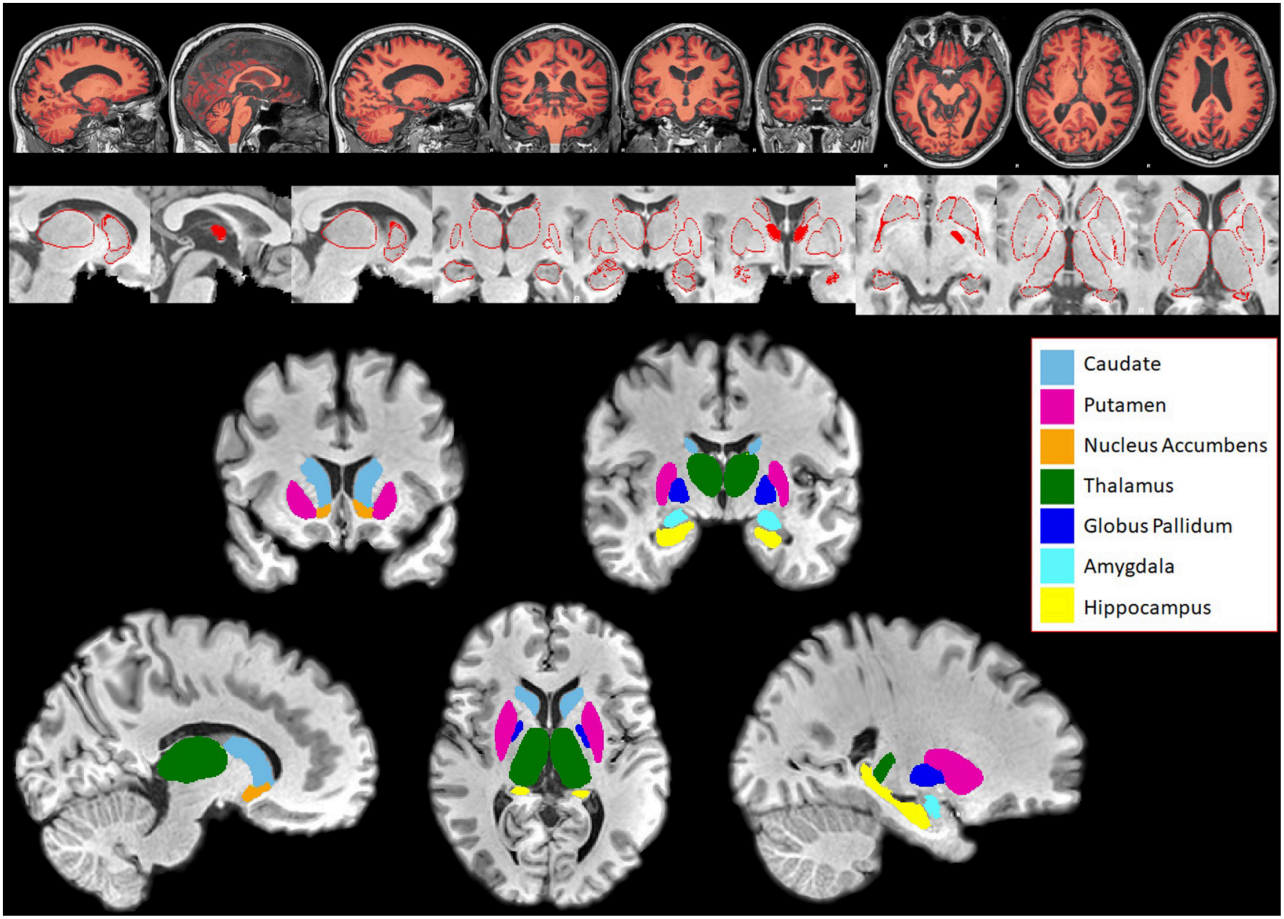
Subcortical structures segmented in Patients and Controls for comparison using FIRST. **Top panel:** Segmentation of white and gray matter generated by SIENAX overlaid on a subject's brain. **Middle panel:** Series of pictures of red outlines that represent segmented subcortical structures overlaid on top of the subject's T1-weighted brain. These pictures are examined for quality assessment purposes. **Bottom panel:** Subcortical structures segmented using FIRST, overlaid on top of the subject's skull stripped T1 weighted brain. Bilateral caudate, putamen, nucleus accumbens, thalamus, globus pallidus, amygdala, and hippocampus were segmented in both patients and controls.

Data analysis was performed on T1 images using FMRIB Software Library (FSL, version 5.0.1) (16). SIENAX (from FMRIB software library) (17) was used to obtain the volumes of normalized white and gray matter as well as the total brain volume. Normalized total brain volume was used as a covariate in volumetric and vertex analysis to control for its effects. FIRST (FSL's Integrated Registration and Segmentation Tool) (18) was used to segment subcortical structures (bilateral thalamus, caudate, putamen, globus pallidus, hippocampus, amygdala, nucleus accumbens) and FSLstats was used to obtain the volume of each subcortical structure. Structures that were segmented for both patients and controls are shown in Figure 1. All subcortical structure volumes were normalized by multiplying each subjects by their respective V-scaling factor. The V-scaling factor, which represents the value each subjects brain were multiplied to register to MNI-152 standard space (19) was obtained using FSL's SIENAX (20).

### Volumetric Analysis

Volumetric Analysis was performed using IBM SPSS software (version 23, IBM Corporation, New York). Pairwise comparisons using ANOVA were used to examine group differences of characteristics. Multivariate analysis of covariance (MANCOVA) was used to evaluate volumetric differences in subcortical structures. Age and normalized total brain volume were significant predictors of variance and were used as covariates. Bonferroni correction with significance at *p* ≤ 0.05 was used to correct for multiple comparisons.

### Vertex Analysis

Vertex analysis was performed using FSL's generalized linear model to construct a statistical matrix and Randomize (16) to perform a non-parametric permutation inference on all the segmented subcortical structures. The test was corrected for multiple comparison at *p* ≤ 0.05, using age and normalized total brain volume as covariates.

### Atlas Overlay

The result of hippocampus vertex analysis was overlaid on top of a hippocampus substructure atlas generated by FreeSurfer (21). The results of thalamus vertex analysis which represent surface reductions were overlaid on top of an Oxford thalamic connectivity probability substructure atlas (22) generated by FSL. Vertex analysis of other subcortical regions were not overlaid due to the lack of other 3D subregional subcortical atlas meshes.

## Results

### Demographic Results

Characteristics of both groups are listed in Table 1. Each alcohol-dependent subject was age-matched with a healthy control. As such, there were no group differences in participant age (*p* = 0.932). Additionally, there were no group differences in years of education (*p* = 0.961). Alcohol-dependent patients had greater lifetime drinking history than healthy controls (*p* = 0.003). Alcohol-dependent patients also scored higher points on AUDIT-K (*p* < 0.001) and SMAST (*p* < 0.001) tests. Healthy controls had greater average weight (*p* < 0.001) than alcohol-dependent patients but did not differ in total brain volume (*p* = 0.145).

**Table 1:** Characteristics of subjects.

**Characteristics**	**Alcohol-dependent Patients Mean (SD)**	**Healthy Controls Mean (SD)**	***p*-value**	**Pairwise comparison**
	***n* = 21**	***n* = 21**		
Age (years)	50.6 (8.2)	50.4 (6.1)	0.932	AD = HC (NS)
Lifetime drinking history	80234 (87986.6)	18530.6 (19141.4)	0.003	AD > HC
Education (years)	13.7 (2.7)	13.7 (3.0)	0.961	AD = HC (NS)
Weight (kg)	63.7 (7.7)	77.3 (10.6)	<0.001	HC > AD
AUDIT-K	28.1 (11.0)	11.2 (9.0)	<0.001	AD > HC
SMAST	20.2 (7.6)	3.1 (6.3)	<0.001	AD > HC

*AUDIT-K, Alcohol Use Disorder Identification Test; SMAST, Short Michigan Alcohol Screening Test; AD, Alcohol-dependent patients; HC, Healthy Controls*.

### Volumetric Results

Cortical and subcortical differences in volumes of the two groups are displayed in Tables 2, 3. MANCOVA showed no significant main effect of group characteristics (age: *p* = 0.109, weight: *p* = 0.249, education: *p* = 0.577, SMAST: *p* = 0.092, AUDITK: *p* = 0.414) on cortical and brain volumes. Additionally, MANCOVA showed that there were no correlations between lifetime drinking history and brain volumes (*p* = 0.392). MANCOVA showed a significant main effect of group (alcohol-dependent patients and healthy controls) on gray matter (*p* < 0.01, corrected) and white matter (*p* < 0.01, corrected), as shown in Table 2, and subcortical volumes with significant differences in the bilateral thalamus (left: *p* < 0.01, right: *p* = 0.01), bilateral putamen (left: *p* = 0.02, right: *p* < 0.01), right globus pallidus (*p* < 0.01), bilateral hippocampus (left: *p* = 0.05, right: *p* = 0.03), and bilateral nucleus accumbens (left: *p* < 0.01, right: *p* < 0.01) as shown in Table 3. There were no significant correlations between lifetime drinking history and the volumetric changes of subcortical structures.

**Table 2:** Total brain volume, gray, and white matter group comparisons.

**Region**	**Alcohol-dependent** **patients Mean (SD)**	**Healthy controls** **Mean (SD)**	***p*-value** **(uncorrected)**	***p*-value (Bonferroni** **corrected)**	**Effect size**	**Pairwise comparison**
	***n* = 21**	***n* = 21**				
Total brain volume (cm^3^)	1417.1 (92.0)	1464.0 (111.5)	0.15			AD = HC (NS)
**Gray matter (cm**^**3**^**)**	**743.6 (48.0)**	**750.2 (48.2)**	**<0.001**	**<0.01**	**0.925**	**HC** **>** **AD**
**White matter (cm**^**3**^**)**	**673.5 (48.1)**	**713.8 (48.2)**	**<0.001**	**<0.01**	**0.904**	**HC** **>** **AD**

*Significant volumetric reductions are in bold*.

**Table 3:** Group-wise differences of subcortical volumes.

**Region**	**Alcohol-dependent** **patients Mean (SD)**	**Healthy controls** **Mean (SD)**	***p*-value** **(uncorrected)**	***p*-value** **(Bonferroni corrected)**	**Effect size**	**Pairwise comparison**
	***n* = 21**	***n* = 21**				
**L thalamus (cm**^**3**^**)**	**7.23 (0.99)**	**8.18 (0.76)**	**<0.001**	**<0.01**	**0.50**	**HC** **>** **AD**
**R thalamus (cm**^**3**^**)**	**7.00 (0.82)**	**7.78 (0.86)**	**0.001**	**0.01**	**0.44**	**HC** **>** **AD**
L caudate (cm^3^)	3.34 (0.45)	3.62 (0.36)	0.055	0.77	0.25	AD = HC (NS)
R caudate (cm^3^)	3.51 (0.46)	3.66 (0.47)	0.172	2.40	0.19	AD = HC (NS)
**L putamen (cm**^**3**^**)**	**4.82 (0.68)**	**5.55 (0.64)**	**0.002**	**0.02**	**0.41**	**HC** **>** **AD**
**R putamen (cm**^**3**^**)**	**5.04 (0.68)**	**5.62 (0.64)**	**<0.001**	**<0.01**	**0.50**	**HC** **>** **AD**
L Globus Pallidus (cm^3^)	1.78 (0.38)	1.96 (0.44)	0.017	0.23	0.31	AD = HC (NS)
**R globus pallidus (cm**^**3**^**)**	**1.79 (0.38)**	**2.06 (0.39)**	**<0.001**	**<0.01**	**0.47**	**HC** **>** **AD**
**L hippocampus (cm**^**3**^**)**	**3.60 (0.73)**	**4.07 (0.58)**	**0.003**	**0.05**	**0.38**	**HC** **>** **AD**
**R hippocampus (cm**^**3**^**)**	**3.85 (0.74)**	**4.36 (0.66)**	**0.002**	**0.03**	**0.39**	**HC** **>** **AD**
L amygdala (cm^3^)	1.25 (0.31)	1.30 (0.25)	0.096	1.34	0.22	AD = HC (NS)
R amygdala (cm^3^)	1.31 (0.35)	1.46 (0.31)	0.247	3.45	0.16	AD = HC (NS)
**L nucleus accumbens (cm**^**3**^**)**	**0.56 (0.15)**	**0.66 (0.12)**	**<0.001**	**<0.01**	**0.52**	**HC** **>** **AD**
**R nucleus accumbens (cm**^**3**^**)**	**0.41 (0.14)**	**0.49 (0.11)**	**<0.001**	**<0.01**	**0.63**	**HC** **>** **AD**

*L, Left; R, Right. Significant volumetric reductions are in bold*.

### Vertex Analysis Results

**Figure 2:**
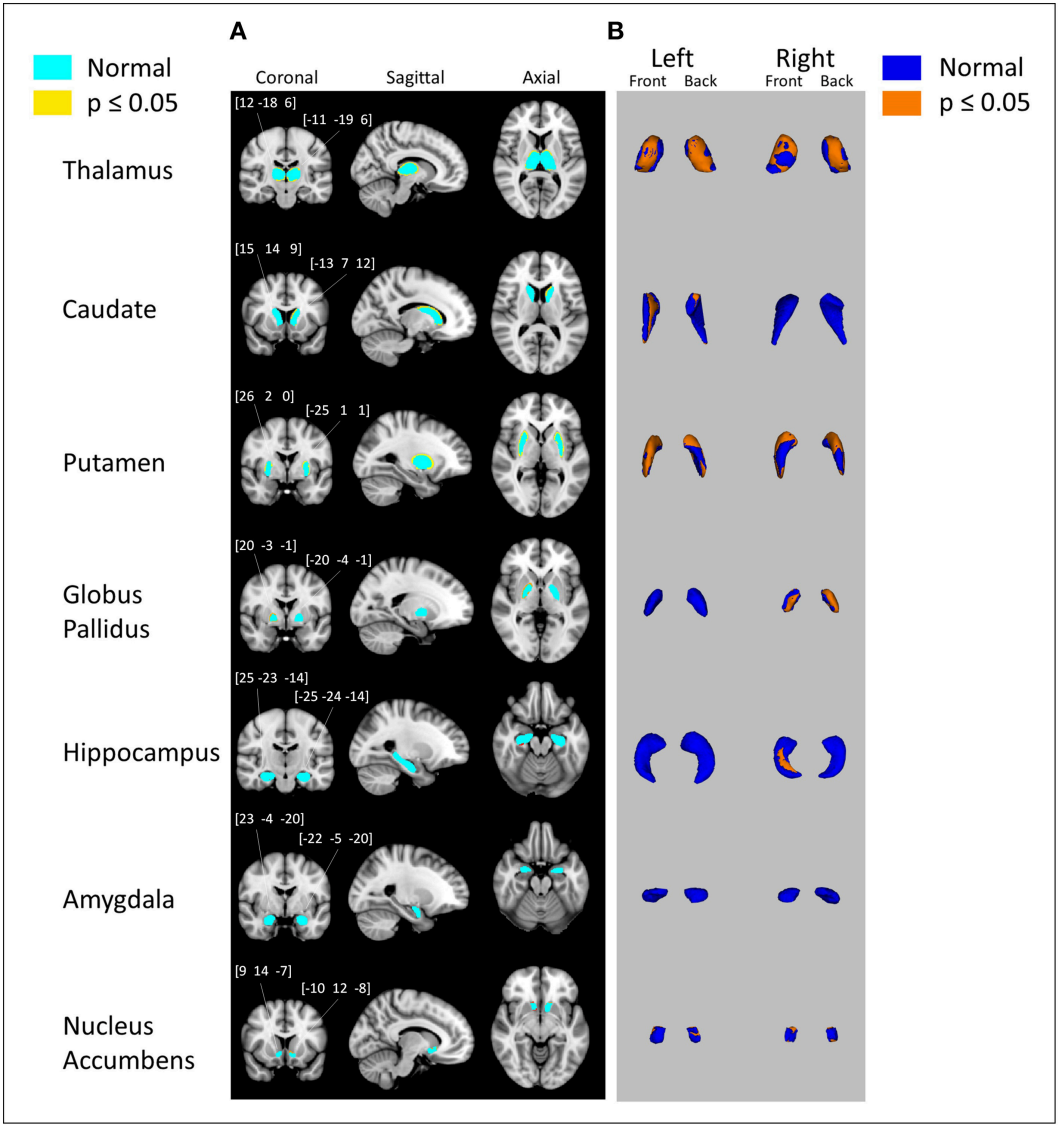
Subcortical surface alterations in thalamus, caudate, putamen, globus pallidus, hippocampus, amygdala, and nucleus accumbens. Vertex analysis adjusted for age, total brain volume; *p* = 0.05 (FDR corrected). **(A)** Coronal, sagittal, and axial views of the results of vertex analysis. Regions in light blue represent the original subcortical structure. Regions in yellow and orange represent the outlines of where volumetric reduction occurred. Numbers above coronal views of subcortical structures represent the MNI coordinates of right and left subcortical structures **(B)** 3D rendering of the results of vertex analysis. Blue models represent the original subcortical structure. Orange layers represent the locations where volumetric reduction occurred.

Vertex analysis displays probabilistically significant (*p* ≤ 0.05) surface deformations of subcortical structures while using age and total brain volume as covariates. Figure 2A shows each individual subcortical structure (light blue) overlaid on top of coronal, sagittal and axial MNI152 space, with yellow areas representing significant reductions. In Figure 2B, each individual subcortical structure is shown in a 3D model, with blue models representing the subcortical structures and orange layers representing significant reductions. Vertex analysis showed significant subcortical volumetric reductions of alcohol-dependent patients in the bilateral thalamus, bilateral putamen, right globus pallidus, right hippocampus, left caudate, and bilateral nucleus accumbens. All the significant reductions revealed from vertex analysis matched the volumetric reductions in all subcortical structures except in the left hippocampus and left caudate.

### Atlas Overlay Results

**Figure 3:**
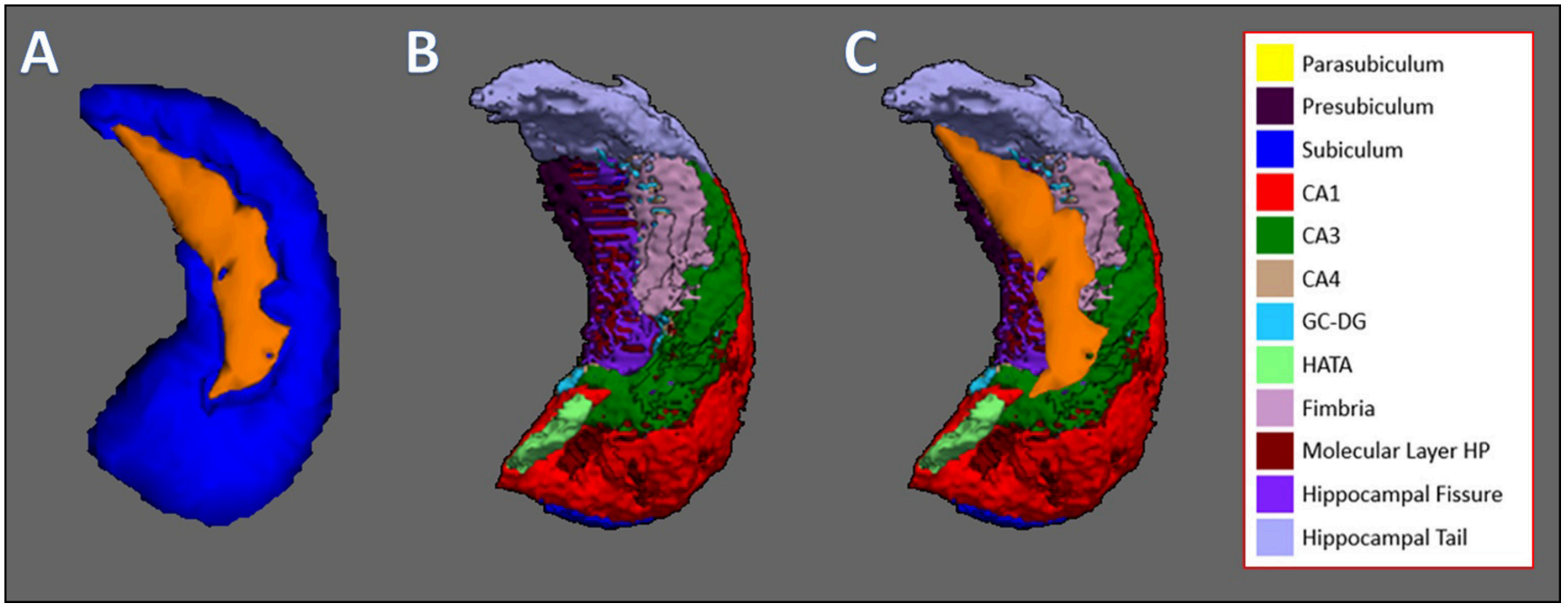
Hippocampus vertex analysis overlaid on top of hippocampal subfields generated by FreeSurfer. **(A)** The result of FSL's vertex analysis of the hippocampus. Orange-colored layer represents the significant reductions of the hippocampus, which is represented by the blue structure. **(B)** Hippocampal subfields generated by FreeSurfer, which are represented by colors: Parasubiculum, yellow; presubiculum, dark purple; subiculum, blue; CA1, red; CA3, green; CA4, brown; GC-DG, light blue; HATA, light green; fimbria, pink; hippocampal molecular layer, dark red; hippocampal fissure, purple; hippocampal tail, light purple. **(C)** Orange layer from vertex analysis is overlaid on top of hippocampal subfields to which subfields experienced volumetric reduction. The subfields that are overlapped by the hippocampal vertex analysis layer are the presubiculum, hippocampal tail, hippocampal molecular layer, hippocampal fissure, fimbria, and CA3.

**Figure 4:**
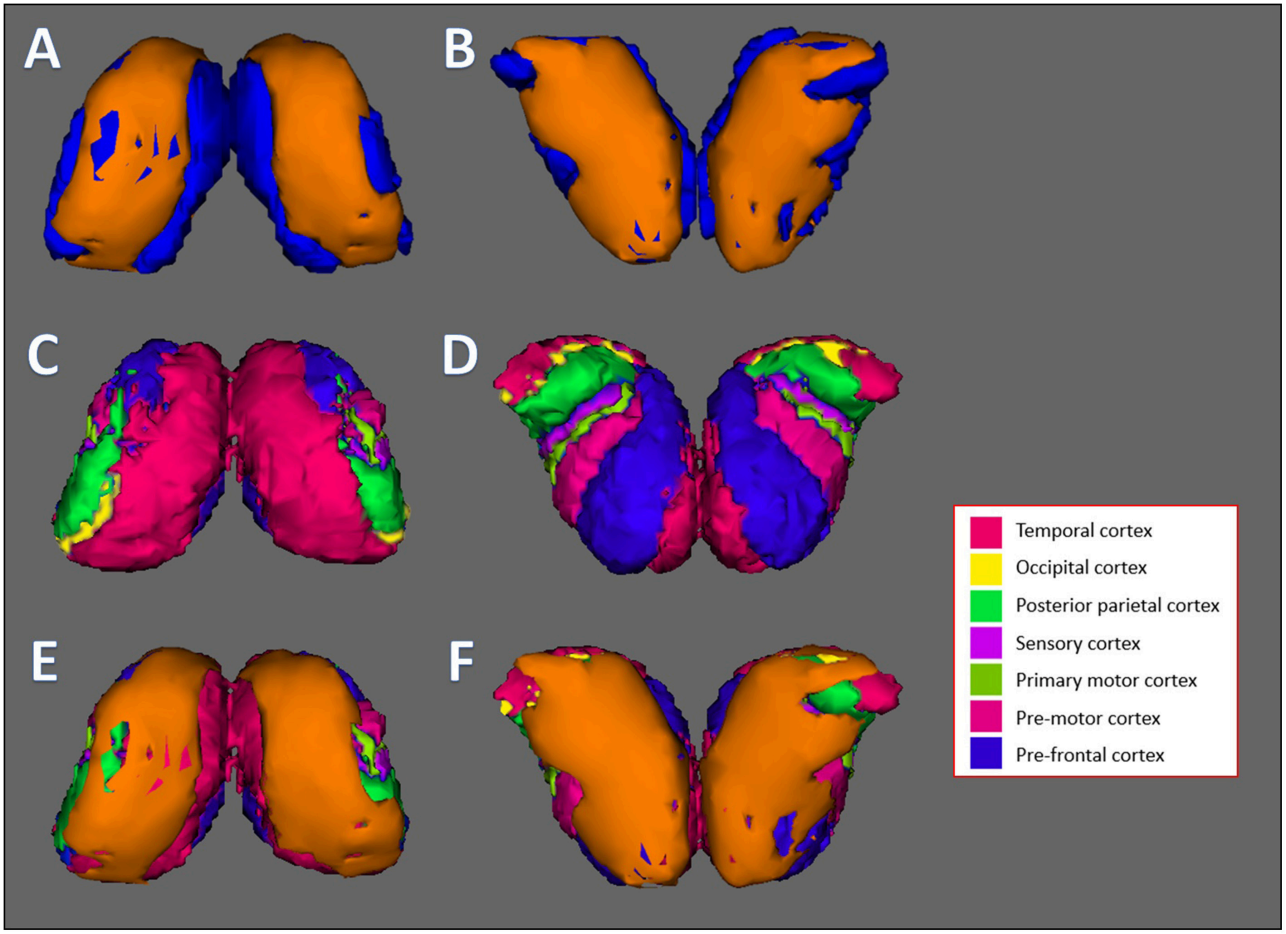
Thalamus vertex analysis overlaid on top of Oxford thalamic connectivity subfields generated by FSL. **(A,B)** Front and back results of FSL's vertex analysis of the thalamus. The orange layer represents the surface where significant reduction of the thalamus occurred. The blue layer represents the original thalamus structure. **(C,D)** The Oxford Thalamic Connectivity Atlas. Each thalamic subfield is represented by a color: Temporal cortex, magenta; occipital cortex, yellow; posterior parietal cortex, green; sensory cortex, purple; primary motor cortex, dark green; pre-motor cortex, pink; pre-frontal cortex, blue. **(E,F)** Front and back results of thalamus vertex analysis, represented by the orange layer, overlaid on top of the Oxford Thalamic Connectivity subfields. Every substructure was overlapped by thalamus vertex analysis layer. **(A,C,E)** represent the front side of the bilateral thalamus while **(B,D,F)** represent the back side.

Figure 3 shows the process of observing specifically which subfield of the hippocampus showed reduction for more clarification. Figure 3A shows the 3D model of the hippocampus vertex analysis (which is derived from results shown in Figure 2B) and Figure 3B shows the FreeSurfer-generated hippocampus model, which is divided into subfields. The orange layer from Figure 3A, which represents the location where significant reduction of the hippocampus occurred, was overlaid on top of hippocampus subfields from Figure 3B to identify which subfields experienced significant reduction. The overlaps between the orange layer and the hippocampal atlas shown in Figure 3C represented reductions in the presubiculum, hippocampus tail, molecular layer hippocampus, hippocampal fissure, fimbria and CA3. Figure 4 shows the same process as shown in Figure 3 but with vertex analysis results (Figures 2B, 4A,B) of the bilateral thalamus and the Oxford Thalamic Connectivity subfields (Figures 4C,E). Overlaps between the thalamic orange vertex analysis layer and the FSL-generated Oxford thalamic connectivity probability atlas (Figures 4E,F) showed reduction in almost every defined thalamic substructure.

## Discussion

For our study, we assessed the volumetric changes of subcortical structures including the hippocampus between two groups: alcohol-dependent patients and healthy control participants. Using the FMRIB automated package on 3T images, we found significant differences in subcortical and cortical volumes between alcohol-dependent patients and healthy controls. Specifically, there were significant reductions in white and gray matter as well as in the subcortical structures: the bilateral thalamus, bilateral putamen, right globus pallidus, bilateral hippocampus and bilateral nucleus accumbens.

There are many factors that could have influenced the outcome of FSL analysis, such as artifacts in MRI images (which were visually inspected and none were found to be drastic) and possible errors in segmentation due to the small size of each subcortical structure (which were also visually inspected using figures shown in Figure 1). However, our analysis using FSL was able to find significant volumetric reductions in similar subcortical structures reported in other studies that also utilized FSL on 1.5T and 3T images. The Fein and Fein study (14) found significant losses in the hippocampus, globus pallidus and nucleus accumbens, with the hippocampus as the largest effect size, and the Grodin study (13) found significant losses in the thalamus, hippocampus and the nucleus accumbens, with the nucleus accumbens as the largest effect size. While the study parameters were not identical, FSL was able to identify reductions in subcortical structures that are common in alcohol-dependent subjects.

Volume reductions of subcortical volumes in alcohol-dependent patients over healthy controls were prominent, as the majority of the volumes we compared had significant reductions. Reductions of subcortical structures in alcohol-dependent patients can be explained by damage to cellular proteins and tissues due to chronic toxic ethanol exposure (23). One particular effect of the damage dealt by alcohol is reduction of cytoskeleton architecture, which results in disrupted cellular trafficking processes (that mediate nutrition transport) and synaptic transmission events (23). In addition, ethanol-induced glucocorticoid elevation disrupts uptake of glucose, which starves subcortical neurons of energy (24). Another factor that can cause damage of brain structures is thiamine deficiency. Thiamine is an essential nutrient required for the processing of enzymes necessary for breakdown of glucose and other pathways that provide brain structures with energy. However, alcohol inhibits the passive uptake of thiamine from consumption, as well as disrupting its role in metabolic pathways, further starving subcortical neurons of nutrients (25, 26).

The largest effect size of volume reductions in subcortical volumes for this study was in the right nucleus accumbens, which was the same subcortical structure reported with the biggest reduction effect size in the Grodin study. Similar reductions in the nucleus accumbens have been reported in studies that observed individuals with alcohol addiction (27). The nucleus accumbens has been attributed to mediating reward-seeking processes and drives alcohol seeking in alcohol-dependent patients (6). One study failed to observe dopamine transporter gene methylation during reward processing in the nucleus accumbens in alcohol-dependent individuals while healthy subjects expressed proper methylation (28). It is likely that such disruption of reward circuitry due to factors caused by alcohol abuse likely contributes to alcohol seeking and withdrawals (29).

Alcohol-dependent patients showed significant volumetric reductions in the thalamus and hippocampus, which was consistent with the results of a study that observed the size of both subcortical structures in patients with Korsakoff syndrome (30). Korsakoff syndrome is a type of amnesia that develops commonly in alcohol-dependent patients with low levels of thiamine, a previously mentioned vitamin that helps the brain generate energy from sugar. Studies suggest that damage to the thalamus and to the pathways connected to the thalamus may contribute to amnesia (31, 32). In addition to amnesia, many studies have reported the involvement of the thalamus and hippocampus in alcohol addiction. Studies showed increased brain activity of alcohol-dependent patients in the thalamus when exposed to alcohol cues (33) and decreased thalamus activation during response inhibition (3). The hippocampus was also shown to activate in alcohol-dependent patients in response to alcohol cues (5) and was shown to display volumetric reductions in alcohol-dependent patients (10).

Alcohol-dependent patients showed significant reductions in both putamen and globus pallidus. Reductions in putamen have been reported in a study that also compared putamen volumes between alcohol-dependent patients and healthy controls (11). Studies have also reported reductions in levels of dopamine receptors (34) and cholinergic receptors in putamen of alcohol-dependent patients (35). The putamen is involved in reward-based learning, and damage to the putamen and its receptors likely contributes to alcohol seeking behavior in alcohol-dependent patients.

In our study, we investigated the locations of each significant volumetric reduction using vertex analysis. As shown in Figure 2, the structures that showed significant reduction through vertex analysis were the bilateral thalamus, left caudate, bilateral putamen, right globus pallidus, right hippocampus, and bilateral nucleus accumbens. In comparison to the MANCOVA results, vertex analysis matched all reductions in subcortical structures except for the left hippocampus, which showed no reductions, and in the left caudate, which showed reductions. This discrepancy is likely due to the different method used for vertex analysis. Vertex analysis utilizes Randomize, a FSL tool for non-parametric permutation inference on neuroimaging data (16), which involves resampling the data (in this case, 5,000 times) to test the null hypothesis multiple times. In order to further clarify the results of vertex analysis, the orange layers of the thalamus and hippocampus (which represent the surface where each subcortical structure experienced significant degradation) were overlaid on top of each subcortical structure's respective atlas. As shown in Figure 4, the vertex analysis layer of the thalamus encompassed some parts of every substructure designated by the Oxford Thalamic Connectivity Atlas (22). As such, it was difficult to identify which specific substructure of the thalamus experienced degradation due to alcohol. It was different for the hippocampus, which had clear overlaps of the hippocampus vertex analysis layer over the substructures designated by the Computational Atlas of the Hippocampus (21). The following hippocampal substructures overlapped with the results of hippocampus vertex analysis: Presubiculum, hippocampal tail, hippocampal molecular layer, hippocampal fissure, fimbria, and CA3. One study that examined changes of hippocampal subfields between alcohol-dependent patients and age-matched controls found similar decreases in the presubiculum and fimbria (36). Several studies on animal hippocampi have shown that the presubiculum and fimbria might contribute to visuo-spatial and episodic memory (37, 38). It is possible that reduction of such hippocampal subfields due to alcohol leads to the impairment of spatial and episodic memory found in alcohol-dependent patients (39, 40).

There are some limitations in this study that should be considered. First, we did not collect data on the family history of alcoholism in subjects, meaning that if there was an association of smaller subcortical structures with likelihood of alcohol addiction, we could not control for the influence of genetics in data. Second, as mentioned previously, the method used for vertex analysis and MANCOVA volumetric analysis are different and yielded conflicting results on two subcortical structures. Third, our study could not find a correlation between lifetime drinking history and the volumetric reductions of subcortical structures. As such, we are unable to test if volumetric reductions of subcortical structures are directly caused by alcohol consumption or if smaller subcortical structures increase the likelihood of alcohol dependence. Fourth, medication data for subjects in this experiment were not collected. It is very well possible that the alcohol-dependent patients recruited from this study were undergoing pharmacological treatment specific to each patient's condition. As such, it is possible that some volumetric reductions observed in this study, as well as lack thereof, are affected by the influence of medication.

In conclusion, our findings were able to find volumetric reductions of subcortical structures in alcohol-dependent patients using FSL, reproducing similar results found in other studies that also utilized FSL. In our results, alcohol-dependent patients had volumetric reductions in both white and gray matter, as well as in subcortical structures such as the bilateral thalamus, bilateral putamen, right globus pallidus, bilateral hippocampus, and bilateral nucleus accumbens. In addition, through vertex analysis, we were able to specify the locations of such reductions on the surfaces of each subcortical structure. We were able to overlay our vertex analysis results on the thalamus and hippocampus subregional atlases and then identified the subfields of the thalamus and hippocampus that showed a reduction in volume. While the thalamus had reductions in all subfields, the hippocampus showed specific reductions in the presubiculum, hippocampal tail, hippocampal molecular layer, hippocampal fissure, Fimbria and CA3, which aligned with previous research that found decreases in similar subfields of the hippocampus in alcohol-dependent patients.

## Author Contributions

J-HS, Y-TK, SK, and H-MB contributed with the study design and data generation. J-HS and Y-TK performed data analysis and interpreted the results. J-HS wrote the manuscript.

### Conflict of Interest Statement

The authors declare that the research was conducted in the absence of any commercial or financial relationships that could be construed as a potential conflict of interest.
